# MODULAR *ANALYTICS*: A New Approach to Automation in the Clinical Laboratory

**DOI:** 10.1155/JAMMC.2005.8

**Published:** 2005

**Authors:** Gary L. Horowitz, Zahur Zaman, Norbert J. C. Blanckaert, Daniel W. Chan, Jeffrey A. Dubois, Olivier Golaz, Noury Mensi, Franz Keller, Herbert Stolz, Karl Klingler, Alessandro Marocchi, Lorenzo Prencipe, Ronald W. McLawhon, Olaug L. Nilsen, Michael Oellerich, Hilmar Luthe, Jean-Luc Orsonneau, Gérard Richeux, Fernando Recio, Esther Roldan, Lars Rymo, Anne-Charlotte Wicktorsson, Shirley L. Welch, Heinrich Wieland, Andrea Busse Grawitz, Hiroshi Mitsumaki, Margaret McGovern, Katherine Ng, Wolfgang Stockmann

**Affiliations:** 1 Department of Pathology Beth Israel DeaconessMedical Center Boston MA 02215-5400 USA; 2 Department of Laboratory Medicine University Hospitals Leuven Leuven 3000 Belgium; 3 Department of Pathology Johns Hopkins Medical Institutions Baltimore MD 21287-0001 USA; 4 Laboratory Services Foundation University Hospitals of Cleveland Cleveland OH 44106 USA; 5 Laboratoire Central de Chimie Clinique Hôpitaux Universitaire de Genève Geneva 1211 Switzerland; 6 Zentrallabor Institut für Klinische Biochemie und Pathobiochemie Universitaetsklinik Wuerzburg Wuerzburg 97080 Germany; 7 Institut für Klinische Chemie der Universität zu Köln Köln 50924 Germany; 8 Department of Laboratory Medicine Niguarda Ca' Granda Hospital Milan 20162 Italy; 9 Department of Pathology The University of Chicago Hospitals and Health System Chicago IL 60637-1470 USA; 10 Fuerst Medical Laboratory Oslo 1051 Norway; 11 Department of Clinical Chemistry Georg-August-Universitaet Goettingen 37075 Germany; 12 Laboratoire de Biochimie Générale Centre Hospitalier Universitaire de Nantes Nantes Cédex 01 44035 France; 13 Laboratorio de Bioquímica Hospital Universitario de Valme Sevilla 41014 Spain; 14 Department of Clinical Chemistry and Transfusion Medicine Institute of Laboratory Medicine Sahlgrenska University Hospital Gothenborg 41345 Sweden; 15 Kaiser Permanente NW Department of Pathology Regional Laboratory Portland OR 97230 USA; 16 Klinikum der Albert-Ludwigs-Universitaet Medizinische Klinik Abt. Klinische Chemie Zentrallabor Freiburg 79106 Germany; 17 Hitachi Ltd Tokyo 100-8220 Japan; 18 Roche Diagnostics GmbH Mannheim 68305 Germany; 19 Roche Diagnostics Indianapolis IN 46250 USA

## Abstract

MODULAR *ANALYTICS* (Roche Diagnostics) (MODULAR *ANALYTICS*, Elecsys and Cobas Integra are trademarks of a member of the Roche Group) represents a new approach to automation for the clinical chemistry laboratory. It consists of a control unit, a core unit with a bidirectional multitrack rack transportation system, and three distinct kinds of analytical modules: an ISE module, a P800 module (44 photometric tests, throughput of up to 800 tests/h), and a D2400 module (16 photometric tests, throughput up to 2400 tests/h). MODULAR *ANALYTICS* allows customised configurations for various laboratory workloads. The performance and practicability of MODULAR *ANALYTICS* were evaluated in an international multicentre study at 16 sites. Studies included precision, accuracy, analytical range, carry-over, and workflow assessment. More than 700 000 results were obtained during the course of the study. Median between-day CVs were typically less than 3% for clinical chemistries and less than 6% for homogeneous immunoassays. Median recoveries for nearly all standardised reference materials were within 5% of assigned values. Method comparisons versus current existing routine instrumentation were clinically acceptable in all cases. During the workflow studies, the work from three to four single workstations was transferred to MODULAR *ANALYTICS*, which offered over 100 possible methods, with reduction in sample splitting, handling errors, and turnaround time. Typical sample processing time on MODULAR *ANALYTICS* was less than 30 minutes, an improvement from the current laboratory systems. By combining multiple analytic units in flexible ways, MODULAR *ANALYTICS* met diverse laboratory needs and offered improvement in workflow over current laboratory situations. It increased overall efficiency while maintaining (or improving) quality.


Journal of Automated Methods & Management in Chemistry, **2005** (2005), no. 1, 8–25
© 2005 Hindawi Publishing Corporation

### MODULAR *ANALYTICS*: A New Approach to Automation in the Clinical Laboratory

Gary L. Horowitz,[1] Zahur Zaman,[2] Norbert J. C. Blanckaert,[2] Daniel W. Chan,[3] Jeffrey A. DuBois,[4] Olivier Golaz,[5] Noury Mensi,[5] Franz Keller,[6] Herbert Stolz,[6] Karl Klingler,[7] Alessandro Marocchi,[8] Lorenzo Prencipe,[8] Ronald W. McLawhon,[9] Olaug L. Nilsen,[10] Michael Oellerich,[11] Hilmar Luthe,[11] Jean-Luc Orsonneau,[12] Gérard Richeux,[12] Fernando Recio,[13] Esther Roldan,[13] Lars Rymo,[14] Anne-Charlotte Wicktorsson,[14] Shirley L. Welch,[15] Heinrich Wieland,[16] Andrea Busse Grawitz,[16] Hiroshi Mitsumaki,[17] Margaret McGovern,[18] Katherine Ng,[19] and Wolfgang Stockmann[18]

*[1]Department of Pathology, Beth Israel Deaconess Medical Center, Boston, MA 02215-5400, USA; [2]Department of Laboratory Medicine, University Hospitals Leuven, 3000 Leuven, Belgium; [3]Department of Pathology, Johns Hopkins Medical Institutions, Baltimore, MD 21287-0001, USA; [4]Laboratory Services Foundation, University Hospitals of Cleveland, Cleveland, OH 44106, USA; [5]Laboratoire Central de Chimie Clinique, Hôpitaux Universitaire de Genève, 1211 Geneva, Switzerland; [6]Zentrallabor, Institut für Klinische Biochemie und Pathobiochemie, Universitaetsklinik Wuerzburg, 97080 Wuerzburg, Germany; [7]Institut für Klinische Chemie der Universität zu Köln, 50924 Köln, Germany; [8]Department of Laboratory Medicine, Niguarda Ca' Granda Hospital, 20162 Milan, Italy; [9]Department of Pathology, The University of Chicago Hospitals and Health System, Chicago, IL 60637-1470, USA; [10]Fuerst Medical Laboratory, 1051 Oslo, Norway; [11]Department of Clinical Chemistry, Georg-August-Universitaet, 37075 Goettingen, Germany; [12]Laboratoire de Biochimie Générale, Centre Hospitalier Universitaire de Nantes, 44035 Nantes Cédex 01, France; [13]Laboratorio de Bioquímica, Hospital Universitario de Valme, 41014 Sevilla, Spain; [14]Department of Clinical Chemistry and Transfusion Medicine, Institute of Laboratory Medicine, Sahlgrenska University Hospital, 41345 Gothenborg, Sweden; [15]Kaiser Permanente NW Department of Pathology, Regional Laboratory, Portland, OR 97230, USA; [16]Klinikum der Albert-Ludwigs-Universitaet, Medizinische Klinik, Abt. Klinische Chemie, Zentrallabor, 79106 Freiburg, Germany; [17]Hitachi Ltd, Tokyo 100-8220, Japan; [18]Roche Diagnostics GmbH, 68305 Mannheim, Germany; [19]Roche Diagnostics, Indianapolis, IN 46250, USA*

*Received 30 June 2004; Accepted 18 August 2004*

MODULAR *ANALYTICS* (Roche Diagnostics) (MODULAR *ANALYTICS*, Elecsys and Cobas Integra are trademarks of a member of the Roche Group) represents a new approach to automation for the clinical chemistry laboratory. It consists of a control unit, a core unit with a bidirectional multitrack rack transportation system, and three distinct kinds of analytical modules: an ISE-module, a P800 module (44 photometric tests, throughput of up to 800 tests/h), and a D2400 module (16 photometric tests, throughput up to 2400 tests/h). MODULAR *ANALYTICS* allows customised configurations for various laboratory workloads. The performance and practicability of MODULAR *ANALYTICS* were evaluated in an international multicentre study at 16 sites. Studies included precision, accuracy, analytical range, carry-over, and workflow assessment. More than 700 000 results were obtained during the course of the study. Median between-day CVs were typically less than 3% for clinical chemistries and less than 6% for homogeneous immunoassays. Median recoveries for nearly all standardised reference materials were within 5% of assigned values. Method comparisons versus current existing routine instrumentation were clinically acceptable in all cases. During the workflow studies, the work from three to four single workstations was transferred to MODULAR *ANALYTICS*, which offered over 100 possible methods, with reduction in sample splitting, handling errors, and turnaround time. Typical sample processing time on MODULAR *ANALYTICS* was less than 30 minutes, an improvement from the current laboratory systems. By combining multiple analytic units in flexible ways, MODULAR *ANALYTICS* met diverse laboratory needs and offered improvement in workflow over current laboratory situations. It increased overall efficiency while maintaining (or improving) quality.

### 1. INTRODUCTION

At the beginning of the 21st century, clinical laboratories are faced with many challenges, including reduced fee schedules, demands for faster turnaround times, diminished numbers of qualified technologists, and requests for larger test repertoires. To meet these challenges, laboratories are relying increasingly on automation.

Traditionally, automating a manual test has allowed for better precision and accuracy, faster turnaround time, and around-the-clock availability. Currently, in most laboratories, many, if not most, samples must be placed on several different automated instruments to complete all of the ordered

Correspondence and reprint requests to Gary L. Horowitz, Department of Pathology, Beth Israel Deaconess Medical Center, Boston, MA 02215-5400, USA; Tel: +1 617 667 3648; Fax: +1 617 667 4533; E-mail: gary_horowitz@caregroup.harvard.edu.

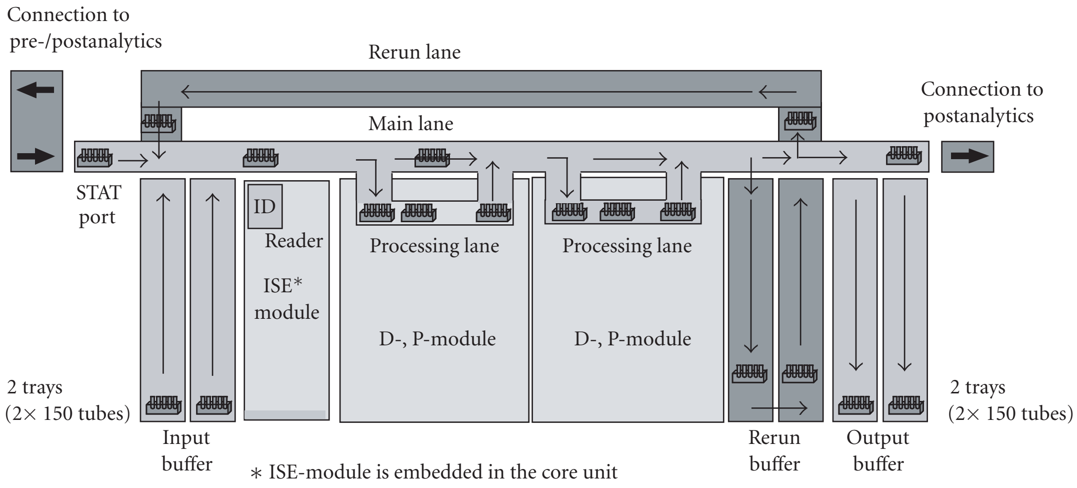

Figure 1: Schematic structure of MODULAR.

tests. Although this represents an advance over manual testing, it is an inherently inefficient process, as each instrument requires its own operators, training courses, reagent systems, maintenance schedules, and proficiency testing.

One approach to enhancing laboratory efficiency has been to attach multiple disparate analysers with a series of conveyor belts or similar transport systems [1]. In these systems, one still has the inefficiency of different instruments (not to mention yet another layer of software) but one gains efficiency from not having to manually transport samples from one instrument to another.

MODULAR *ANALYTICS* from Roche Diagnostics GmbH, Mannheim, Germany, hereafter MODULAR, represents a different approach to automation. By assembling multiple analyser modules with standardised dimensions and interfaces, MODULAR acts more like a single analyser, even though it can be customised by the choice of modules used, the number of modules used, and the specific analytes placed on each module. MODULAR consists of a control unit, a core unit, and analytical modules. The control unit is a Microsoft Windows NT®-based personal computer (PC), from which a single operator can control the entire system. The core unit consists of a bidirectional multitrack transportation system (BMTS) together with a loader/unloader and a rerun buffer. The BMTS is a unique feature of MODULAR, consisting of a main lane, processing lane, and rerun lane, that eliminates queuing of sample racks as they travel between analytical modules. As indicated in Figure 1, sample racks, containing up to five tubes each, are conveyed to modules by the main lane, where they can be transferred to the processing lane. After the sampling process, the rack is returned to the main lane and then conveyed to the next module or to the rerun buffer. The rack remains in the rerun buffer until all test results for those samples are available, at which time the rack is transported either to the unloader or back to the modules where reruns are needed.

We evaluated three kinds of analytical modules: an electrolyte module (ISE900), an 800 tests/h maximum throughput photometric module with an on-board capacity of 44 tests (P800), and a 2400 tests/h maximum throughput photometric module with an on-board capacity of 16 tests (D2400) (abbreviated to ISE-, P-, and D-module, resp., in the following text). The main specifications are presented in Table 1. As noted earlier, MODULAR can be configured with analytical modules in several different ways (e.g., ISE + P + D, ISE + P + P, etc.). In addition, a large number of different chemistries can be placed on the photometric modules (examples are shown in Table 2). Because of the number of on-board chemistries available per module as well as the breadth of this test repertoire, MODULAR can process most serum tests and thereby eliminate the need for separate laboratory classifications such as clinical chemistry, immunology, and therapeutic drug monitoring.

### 2. MATERIALS AND METHODS

This study consisted of two parts: detailed analytical performance experiments at five sites, followed by functionality and practicability experiments at all 16 sites, including hardware evaluation, software evaluation, and chemistry interactions during simulated routine operating conditions. For most sites, the standard MODULAR configuration was one ISE-module, one D-module, and one P-module. MODULAR reagents and calibrators were supplied by Roche Diagnostics in system packs containing bar coded bottles. Imprecision and quality control studies were performed with lyophilised control sera from Roche Diagnostics and control urines from BioRad (BioRad Laboratories, Irvine, Calif, USA). Standardised reference materials were obtained from the National Institute of Standards & Technology (NIST, Washington, DC, USA) and from the Community Bureau of Reference (Brussels).

Table 1: Main specifications of MODULAR *ANALYTICS*.

| Items | | Specification |
|---|---|---|
| Method | | Discrete method of simultaneous analysis for multiple tests according to analyser module combinations |
| Method of sample loading | | Continuous loading of five-position racks |
| Number of batches for racks | | 300 samples (in 2 trays) |
| Rack processing method | | Distribution method in which the racks are captured by the various analyser modules as determined by the Intelligent Process Manager. Intelligent process management ensures most efficient operation, whereby racks are processed in serial, parallel, or serial/parallel mode with full by-pass function and automatic rerun |
| Number of items for analysis | | Maximum of 100 items: photometric (86 tests) + calculation test (8 tests) + blood serum indexes (3 tests) + electrolyte (3 tests) |
| Assay method | | 1-point end, 2-point end, 3-point rate, 3-point, rate A, rate B |
| Calibration | | Linear, k-factor, isozyme, nonlinear methods<br>Nonlinear function = maximum of 6 points<br>4-parameter logit-log, 5-parameter logit-log, 5-parameter exponential function, spline function, polygonal line working curve |
| Monitoring functions | | Such as reaction process monitoring, data review, working curve, and calibration rates |
| Quality control | | Real-time quality control, quality control for samples within a day and between days |
| Retesting function | | Automatic and manual retests are available |
| Control unit | | Windows NT based user interface, touch screen and mouse operation, remote diagnostic access |
| Number of tests for simultaneous analysis | D<br>P<br>ISE | Maximum of 16<br>Maximum of 44 tests<br>Maximum of 3 tests (Na, K, Cl) |
| Processing capability | D<br>P<br>ISE | Maximum of 2400 tests/h<br>Maximum of 800 tests/h<br>Maximum of 900 tests/h |
| Sample pipetting volume | D<br>P<br>ISE | 2∼20 $\mu$L/test (in 0.1 $\mu$L steps)<br>2∼35 $\mu$L/test (in 0.1 $\mu$L steps)<br>15 $\mu$L/test |
| Reagent pipetting and reaction volume | D/P | 20∼270 $\mu$L/test (in 1 $\mu$L steps) reagent pipetting, 180∼380 $\mu$L reaction volume |
| Reaction disk | D<br>P | Turntable method, each 240 reaction cuvettes inside and outside circumferences<br>Turntable method, 160 reaction cuvettes |
| Reaction time | D<br>P | 10 min<br>1∼10 min (in 1 min steps) |
| Photometer | D/P | Concave diffraction grating multiwavelengths photometer (12 wavelengths), 0∼3 ABS (2 units on D-module) |

The protocols for the detailed analytical performance experiments in general followed the ECCLS and NCCLS guidelines [2, 3] and are summarised in Table 3 [4, 5, 6, 7, 8, 9]. The instruments used for comparison purposes were mainly Roche/Hitachi 747 (in three laboratories) and Roche/Hitachi 917 (in two laboratories). In all, as indicated in Table 2, 34 analytes covering 45 different methods were tested, with representative assays for all analyte groups from the manufacturer's available test menu.

The protocols for functionality and practicability experiments are summarised in Table 4 [10, 11]. These studies focused on precision while running a normal workload, comparisons to existing methods, and practicability as assessed by a detailed questionnaire. In addition, some laboratories undertook detailed workflow studies. Up to 40 analytes were processed at each site, encompassing a total of 65 different analytes and 81 different methods.

Table 2: Analyte selection.

| Enzymes | | Protocol | Proteins | | Protocol |
|---|---|---|---|---|---|
| ALP | Alkaline phosphatase AMP | a & b | A1M | $\alpha_1$-Microglobulin TIA | a & b |
| ALPO | Alkaline phosphatase DGKCh | b | ALBU | Albumin in urine TIA | a & b |
| ALT | Alanine aminotransferase IFCC | b | ASLO | Antistreptolysin O LPIA | b |
| AMYL | Amylase total liquid EPS | a & b | B2M | $\beta_2$-Microglobulin TIA | b |
| PAMY | Amylase pancreatic liquid EPS | b | CRP | C-reactive protein TIA | a & b |
| AST | Aspartate aminotransferase IFCC | a & b | FERRI | Ferritin LPIA | a & b |
| CHE | Cholinesterase Butyryl | b | GPROT | $\alpha_1$-Glycoprotein TIA | b |
| CK | CK NAC act | a & b | HBA1c | Glycated Haemoglobin TIA | a & b |
| CK-MB | Creatine kinase MB | a & b | HGLOB | Haptoglobin TIA | b |
| GGT | $\gamma$-Glutamyl transferase Szasz | b | IGA | Immunoglobulin A, TIA | a & b |
| LD | Lactate dehydrogenase (L→P) | b | IGG | Immunoglobulin G, TIA | a & b |
| LD-1 | Lactate dehydrogenase isoenzyme 1 | b | IGM | Immunoglobulin M, TIA | a & b |
| LDHO | Lactate dehydrogenase DGKCh | a & b | MYO | Myoglobin TIA | a & b |
| LDHS | Lactate dehydrogenase SFBC | b | RF | Rheumatoid factor LPIA | b |
| LIP | Lipase colorimetric | a & b | TRANS | Transferrin TIA | b |
| | | | U/CSF | Protein in urine/CSF turbidim | b |
| **Substrates/electrolytes** | | **Protocol** | **TDM/others** | | **Protocol** |
| ALB | Albumin BCG (plus) | a & b | CARB | Carbamazepine Cedia | b |
| CHOL | Cholesterol CHOD-PAP | a & b | DIG | Digoxin LPIA | a & b |
| CRE+ | Creatinine enzymatic (plus) | a & b; c | GENTA | Gentamicin Cedia | b |
| CREJ | Creatinine Jaffé | a & b; c | NAPA | N-acetyl-procainamide Cedia | b |
| DBIL | Bilirubin direct Jendrassik | b | PHEBA | Phenobarbital Cedia | b |
| TBIL | Bilirubin total DPD | b | PHENY | Phenytoin Cedia | b |
| ETH | Blood alcohol ADH | b | PROCAI | Procainamide Cedia | b |
| FRUC | Fructosamine | b | SALY | Salicylate Iron complex | b |
| GLUK | Glucose HK | a & b | THEO | Theophylline Cedia | a & b |
| GLUP | Glucose GOD-PAP | b | TOBR | Tobramycin Cedia | b |
| HDL | HDL cholesterol liquid | a & b | VALP | Valproic acid Cedia | b |
| LDL | LDL cholesterol liquid | b | AT III | Antithrombin III | b |
| LACT | Lactate w/o deproteinization colorimetric | b | T4 | Thyroxine Cedia | b |
| $NH_3$ | Ammonia UV | b | T-UP | T-Uptake Cedia | b |
| TG | Triacylglycerol GPO-PAP | a & b | | | |
| TP | Total protein Biuret | a & b | | | |
| UA | Uric acid PAP | a & b; c | | | |
| UIBC | Unsaturated iron binding capacity | b | | | |
| UREA | Urea (BUN) kinetic UV | a & b; c | | | |
| CA | Calcium OCPC | a & b; c | | | |
| $CO_2$ | Bicarbonate kinetic UV | a & b | | | |
| FE | Iron ferrozine | a & b | | | |
| MG | Magnesium xylidyl blue | a & b | | | |
| PHOS | Phosphorus molybdate, UV | a & b; c | | | |
| Na, K, Cl | Sodium, potassium, Chloride; indirect ISE | a & b; c | | | |

a: analytical performance protocol (45 methods for 34 analytes).
b: functional performance and practicability protocol (81 methods for 65 analytes).
c: two applications (serum/plasma and urine).

At the evaluators' first meeting, a set of expected performance criteria were agreed upon (Table 5). CV limits were defined for groups of analytes at concentrations near the medical decision level. The criteria for imprecision were designed to take into account state-of-the-art performance, routine service requirements of the laboratory, and statistical error propagation [12].

The study was supported by CAEv, a program for "Computer Aided Evaluation" [13], which allows the definition of protocols, the sample and test requests for online (and offline) data capture, and statistical evaluation of the results. Data were validated by the evaluators and transferred electronically to the central study organisation at Roche Diagnostics in Mannheim, Germany.

Table 3: Evaluation protocol of the analytical performance study.

| | |
|---|---|
| Imprecision | *Within-run*<br>Performed on three days, each day one run with 21 aliquots. Two control materials (serum, urine) with different concentrations of the analyte and one human specimen pool at the diagnostic decision level were used. The methods tested were ALP, AMYL, AST, CK, LDH, LIP, ALB, CHOL, CREA J, CREA+, GLU, TG, TP, UA, UREA, CA, $CO_2$, FE, MG, PHOS, and CRP on both D- and P-modules; HDL, CK-MB, FERRI, MYOGB, IGA, G, M, HBA1C, DIGOX, THEO in serum/plasma and A1M, ALB, CA, CREA+, CREA J, PHOS, UA, UREA in urine on P-module only; NA, K and CL in serum/plasma and urine on the ISE-module<br>*Between-day*<br>Two control materials with different concentrations of the analyte, over 21 days were used. Precision is derived from the second of triplicate measurements. The methods investigated were the same as for the within-run experiments<br>*Functional sensitivity [4]*<br>Three serum pools were diluted to five different concentration levels of the analyte which were aliquoted to ten samples and stored at $-20^{\circ}$C. The concentrations of the aliquots were determined over ten days in triplicates. Methods investigated: Ferritin on P-module only |
| Drift | Two control sera and the calibrator were determined every half an hour during eight hours, and then in addition after 24 hours on D-module for selected analytes ($CO_2$, CA, FE, and CRP) to confirm the stability in the reagent lines. At zero hour the base value was determined as the median of triplicate measurements. The percentage recovery from the base value was taken as the measure for drift effects. The drift behaviour was tested with 11 methods on D- and P-modules: AST, CK, CHOL, CREA J, GLUC-HK, TP, UA, $CO_2$, CA, FE, and CRP, two methods on the P-module only: DIG, THEO, and three methods on the ISE-module: NA, K, and CL |
| Analytical range limits | *Protocol is based on [5]*<br>Mixing of a high-level with a low level specimen led to a dilution series of 11 concentration steps with nine dilution steps plus two basic concentrations. Triplicate measurements of samples from the 11 concentration steps were performed and the median for each step was calculated. The regression line (Passing/Bablok regression [6]) was calculated using values of five concentrations, the range of which was assumed to be linear. The target values for all concentration steps were calculated from the regression lines<br>Methods investigated: AST, CK, CHOL, CREA J, GLU, TP, UA, CA, $CO_2$, FE, and CRP on both D- and P-modules; HDL, FERRI, MYOGB, IGA, G, M, in serum/plasma and A1M, ALB, CA, CREA+, CREA J, PHOS, UA, UREA in urine on P-module only; NA, K, and CL in serum/plasma and urine on the ISE-module |
| Carry-over | *Sample related*<br>*Model of Broughton [7]*<br>Measurements of three aliquots of a high-concentration sample ($h_1 \cdots h_3$) were followed by measurements of five aliquots of a low-concentration sample ($l_1 \cdots l_5$). This series was repeated 10 times. If a carry-over effect exists, $l_1$ is the most influenced, $l_5$ the least influenced aliquot. The sample-related carry-over—median ($l_1 - l_5$)—was compared with the imprecision of the low-concentration sample. Methods investigated: CK and ferritin (analytes having a wide physiologic range) and urine versus serum for creatinine and albumin<br>*Reagent dependent [8]*<br>Assay A influences assay B<br>Carry-over caused by the cuvettes was tested between the triglycerides and lipase assays; the lipoprotein lipase of the triglycerides assay shows lipase activity.<br>Test A was pipetted into 21 cuvettes and the analyser was stopped. Assay B was performed in 42 cuvettes; the first 21 determinations might be influenced by assay A, the last 21 determinations were uninfluenced. The difference of the medians of both series is the carry-over<br>Carry-over caused by reagent probes and stirrers was tested between the triglycerides and lipase assays and between a one molar phosphate buffer (this is approximately a tenfold higher concentration than is usually used in the reagents) and the phosphate assay<br>Assay B was carried out 21 times. In a second step tests A and B were requested 21 times. The carry-over was the difference between the medians of both series. The carry-over effects were compared with the imprecision and the diagnostic relevance of assay B |

Table 3: Continued.

| | |
|---|---|
| Interference | *Protocol according to Glick [9]* |
| | A serum with concentrations at the relevant decision level was spiked with the interfering substance and a dilution series of ten dilution steps was prepared with the same baseline serum. The different analytes were measured in triplicates. The concentration of the interfering substance was related to the serum index of the instrument. The percentage recovery of the baseline value from the corresponding analyte was calculated for each dilution step |
| | The methods tested were ALP, AST, CK, ALB, CHOL, CREA J, CREA+, GLU, TP, UA, UREA, CA, $CO_2$, FE, MG, PHOS, NA, K, CL for conjugated and unconjugated bilirubin; AST, CK, CHOL, HDL, CREA J, CREA+, GLU, TP, UA, CA, FE, NA, K, CL CRP, FERRI, MYO, IGA, G, M, DIGOX, THEO for lipaemia, and AMYL, AST, CK, LDH, LIP, CHOL, CREA J, GLU, TG, TP, UA, CA, FE, NA, K, CL for haemolysis |
| Accuracy | *Interlaboratory survey* |
| | Two control materials with concentrations not known to the evaluators were used for AST, CK, ALB, CHOL, CREA J, CREA+, GLU, TP, UA, UREA, CA, FE, MG, PHOS, NA, K, and CL. The assigned values for several substrate methods were related to reference methods. The median was calculated from the second of triplicate measurements over five days |
| | Standard reference materials (CRM, NIST) for certain enzyme, substrate, and electrolyte methods were analysed on one day in triplicate measurements. The methods tested were CHOL, CREA J, CREA+, UA, UREA, CA, MG, NA, K, and CL in NIST material and AST and CK in the CRM material |
| Method comparison | Five to fifteen fresh human specimens depending on analytes were measured each day for 10 days on MODULAR and on the comparison instruments. The specimens covered as much of the analytical range as possible. The methods were compared by calculation of the Passing/Bablok regression line [6] |
| | The methods tested were the same as for the within-run experiment |

Table 4: Evaluation protocol of functionality and practicability.

| | |
|---|---|
| Routine simulation [10] | *Precision in a simulated routine run* |
| | The first of these two experiments tests for potential systematic or random errors by comparing the imprecision of the reference results (standard batch, $n = 15$) with that of results from samples run in a pattern simulating routine sampling (randomised sample requests, $n > 10$). The randomised sample requests were simulated in CAEv according to each laboratory's routine sampling pattern. The samples were control materials or patient sample pools |
| | The second of the two experiments processed at each site included "provocation steps" designed to interrupt the smooth flow of work. These actions included deliberately running low on reagent, introducing samples with insufficient volume, and forcing bar code read errors |
| Practicability | Practicability was assessed using a questionnaire with approximately 200 questions covering all important attributes of an analytical system [11] |
| | The assessment of each attribute was rated according to a scale from 1 to 10. A rating of 1 was defined as unimportant, useless, or poor; a rating of 10, absolutely necessary or excellent; a rating of 5, acceptable or comparable to the present laboratory situation |
| Workflow | The participating laboratories in the workflow study configured MODULAR according to their specific needs. The primary goal for each laboratory was to examine whether MODULAR would meet their requirements for routine use in their laboratory. Routine workloads were replicated and reprocessed on MODULAR using CAEv to capture the requests either directly from the routine analysers or via a download from the LIS. In lab A, the sample rack processing time (sample rack placement on MODULAR to results available) was measured with samples arriving at the MODULAR in real time during a routine working day. In lab B, a 24-hour workload was processed as a single large batch, then again as multiple smaller batches (real-time processing). Lab B also characterised samples processed through automatic rerun, measured the sample processing time (equal to rack processing time for STAT samples) when various STAT samples were introduced through the STAT sample port during the morning workload, and examined maintenance protocols for maximising MODULAR operation time and operator convenience. Lab C challenged a PP configuration by continuously loading and processing approximately 1500 samples with requests for 40 different analytes in one run |

### 3. RESULTS

### 3.1. Imprecision

The within-run coefficient of variation (CV) for nearly all methods of enzymes, substrates, and electrolytes was below 2%, with typical CVs of 1%. For specific proteins, drugs, and urine analytes, typical within-run CVs were between 1% and 3%. Within-run imprecision on D- and P-modules was comparable. One specific set of experiments allowed for the comparison of imprecision for tests run in a batch mode versus tests run in a random access mode (Table 4, "Precision in a simulated routine run"). When compared to a standard batch run, one would expect imprecision to be higher in a run designed to simulate routine working conditions (i.e., in which many analytes are run, on many samples, in a random access mode). As shown in Figure 2, the CVs obtained on MODULAR in the random request part were only slightly higher than in the batch part. Of particular note is the fact that the results for most of the enzyme and substrate methods were produced by two distinct modules at each site. As an example, in one laboratory using a P + P configuration, the calcium CV on each module was approximately 1%, but the overall (combined) CV was 2.3% because of a difference in the median values from the modules (nearly 5%). The between-day CVs taken as the median from the five laboratories were below 3%. Typical CVs were 1% to 2% for the enzymes, substrates, and electrolytes, and 1% to 4% for the specific proteins, drugs, and urine methods. Of all the analytes, only bicarbonate with a CV of 7.2% exceeded the performance criteria (3%).

As an additional quality indicator of imprecision, one laboratory determined the functional sensitivity for the ferritin assay; the corresponding precision profile is shown in Figure 3. Functional sensitivity is defined as the concentration at which the between-day CV reaches 20% [4]. At the manufacturer-defined lower detection limit of 15 $\mu$g/L (or 5 $\mu$g/L, using the increased sample volume rerun feature), the between-day CV was just 14% (or 12%).

### 3.2. Drift

With the exception of bicarbonate, no drift effects were observed in any of the 16 methods tested over an eight-hour period. Bicarbonate showed a drift over eight hours of approximately 5% (the decline was less than or equal to 2 mmol/L). For all four analytes selected to test drift after 24 hours without additional priming on the D-module, the recovery was between 95 and 105%.

### 3.3. Analytical range limits

The manufacturer's claims for linearity ranges were verified, to the extent possible, for the methods tested in serum and urine as indicated in Tables 3 and 6. Linearity on D- and P-modules were comparable.

### 3.4. Carry-over

Sample-related carry-over [7] was tested on P-module with analytes having a wide physiologic range (CK and ferritin) and with urine versus serum for creatinine and albumin. The ratio for the high and low serum analytes was 200:1; for urine/serum creatinine, 140:1; for serum/urine albumin, 10 000:1. No significant carry-over effect as defined by the expected performance criteria was observed when the difference from the first to the fifth sample was compared to the imprecision of the method.

Because MODULAR P- and D-modules depend on reusable cuvettes, probes, and stirrers for analysis, we also looked for evidence of reagent-dependent carry-over [8]. There was no relevant reagent-dependent cuvette carry-over (lower than twofold standard deviation) observed between the triglycerides and lipase assays. When "evasion" (a feature which prevents carry-over by preprogrammed additional washing of probes and stirrers between pipetting of specified tests) was activated as recommended by the manufacturer, reagent-dependent carry-over caused by the reagent probes or the stirrers could not be detected between triglycerides and lipase. No phosphate carry-over was observed.

### 3.5. Interferences

Up to a concentration of 1000 mg/dL of Intralipid, none of the 18 methods tested for lipaemia interference showed a bias of more than 10% (the expected performance criterion). From the 19 methods tested with bilirubin, four methods yielded interferences of more than 10%: cholesterol (220 $\mu$mol/L), enzymatic creatinine (550 $\mu$mol/L), magnesium (340 $\mu$mol/L), and total protein (430 $\mu$mol/L). From the 16 methods tested with haemoglobin, seven methods showed interferences: AST, LDH, and potassium at low haemoglobin concentrations (<50 mmol/L); the other four at higher concentrations: CK (120 mmol/L), iron (120 mmol/L), triglycerides (250 mmol/L), and lipase (235 mmol/L, the latter on D-module only).

### 3.6. Accuracy

Three procedures were used to establish comparability among the five participating laboratories and to assess accuracy. First, as indicated in Table 3, two control sera from the manufacturer were distributed. Ten of the assigned values were established by reference methods used by the German Society of Clinical Chemistry; the values were unknown to the participating laboratories. From all 17 methods tested, the median recoveries were within the accepted range of 95% to 105%. Second, for the standard reference materials (CRM for enzymes and NIST for substrate and electrolyte methods), nine of the ten methods tested were within 5% of the target values; the median recovery for cholesterol was 106%.

Third, a total of 149 method comparisons were done. A condensed version of the method comparisons was obtained by plotting the slopes (ordinates) versus the intercept in percent of the upper medical decision level (abscissas). Few methods exceeded 5% (the acceptance criteria) on any axis. Figure 4a shows the comparisons of the D- versus P-modules; 54 of 57 methods (all but lipase, creatinine, and CRP) met the acceptance criteria. Figure 4b shows the comparisons of the enzyme, substrate, and electrolyte data from the P-module versus the laboratories' routine methods; 50 of 72 methods met the acceptance criteria. Deviations above

Table 5: Expected performance criteria.

| Quality characteristic | Expected performance | |
|---|---|---|
| Imprecision at the medical decision level | *Within-run CVs:* | |
| | enzymes and substrates | 2% |
| | ISE | 1% |
| | specific proteins, therapeutic drugs, drugs of abuse and general chemistries in urine | 4% |
| | *Between-day CVs:* | |
| | enzymes and substrates | 3% |
| | ISE | 2% |
| | specific proteins, therapeutic drugs, drugs of abuse and general chemistries in urine | 6% |
| Imprecision routine simulation | *CV deviations from reference (batch) to random (simulation) part:* | |
| | enzymes/substrates | ΔCV ± 1.0% |
| | ISE | ΔCV ± 0.5% |
| | proteins/drugs/urine methods | ΔCV ± 2.0% |
| Drift | Systematic deviation from the initial value less than 5% | |
| Analytical range limits | Manufacturer claims must be fulfilled<br>Differences between the measured and target values from the dilution series are below 5%<br>In the low concentration range the absolute differences are judged with respect to the diagnostic relevance | |
| Carry-over | Less than 2 standard deviations of within-run imprecision or less than 5% of the diagnostic decision level | |
| Interference | Deviation between baseline and measured value less than 10% [9] | |
| Recovery of assigned value in control materials | *Deviation from the assigned value:* | |
| | for enzymes, substrates, and ISE | ≤5% |
| | for proteins/drugs/urine methods | ≤10% |
| Method comparison | *Slope:* | |
| | deviation from identity line | ≤±5% (10%) |
| | *Intercept:* | |
| | deviation from diagnostic decision level<br>(values in brackets for proteins/drugs/urine methods) | ≤±5% (10%) |
| | *Scatter around the regression line:* | |
| | median distance at the percentile 95 (md95) [14] | |
| | deviation from diagnostic decision level | ≤10% |
| | *The ISE-methods should not differ by more than 5% in the concentration range:* | |
| | 120–180 mmol/L (Na)<br>2–9 mmol/L (K)<br>80–130 mmol/L (Cl). | |

5% on at least one axis were found for t-amylase, AST, CK, CK-MB (activity), lipase, cholesterol, creatinine-Jaffe, glucose, HDL cholesterol, uric acid, calcium, bicarbonate, iron, magnesium, chloride, and sodium (see Table 7 [14, 15, 16]). Figure 4c shows the comparisons of the urine and homogeneous immunoassay methods for the P-module versus the laboratories' routine methods; 15 of 20 methods (all but ferritin, HbA1c, IgM, myoglobin, and theophylline) met the acceptance limits.

The scatter around the regression line, expressed as median distance 95 (md95) [17], was acceptable in most comparisons. Of the 92 comparisons done versus non-MODULAR methods, 13 yielded an md95 greater than 10% of the diagnostic decision level (ALP, CK-MB, lipase, creatinine-Jaffe, creatinine-enzymatic, phosphate, $\alpha_1$-microglobulin, CRP, ferritin, haemoglobin A1c, myoglobin, digoxin, albumin in urine).

### 3.7. Functionality and practicability

Over all laboratories, the routine simulation experiments included approximately 15 500 samples and produced 114 000 test results. Increased imprecision of the results in the randomised phase (Table 4, "Precision in a simulated routine run"), taken as one measure of functionality, was slightly

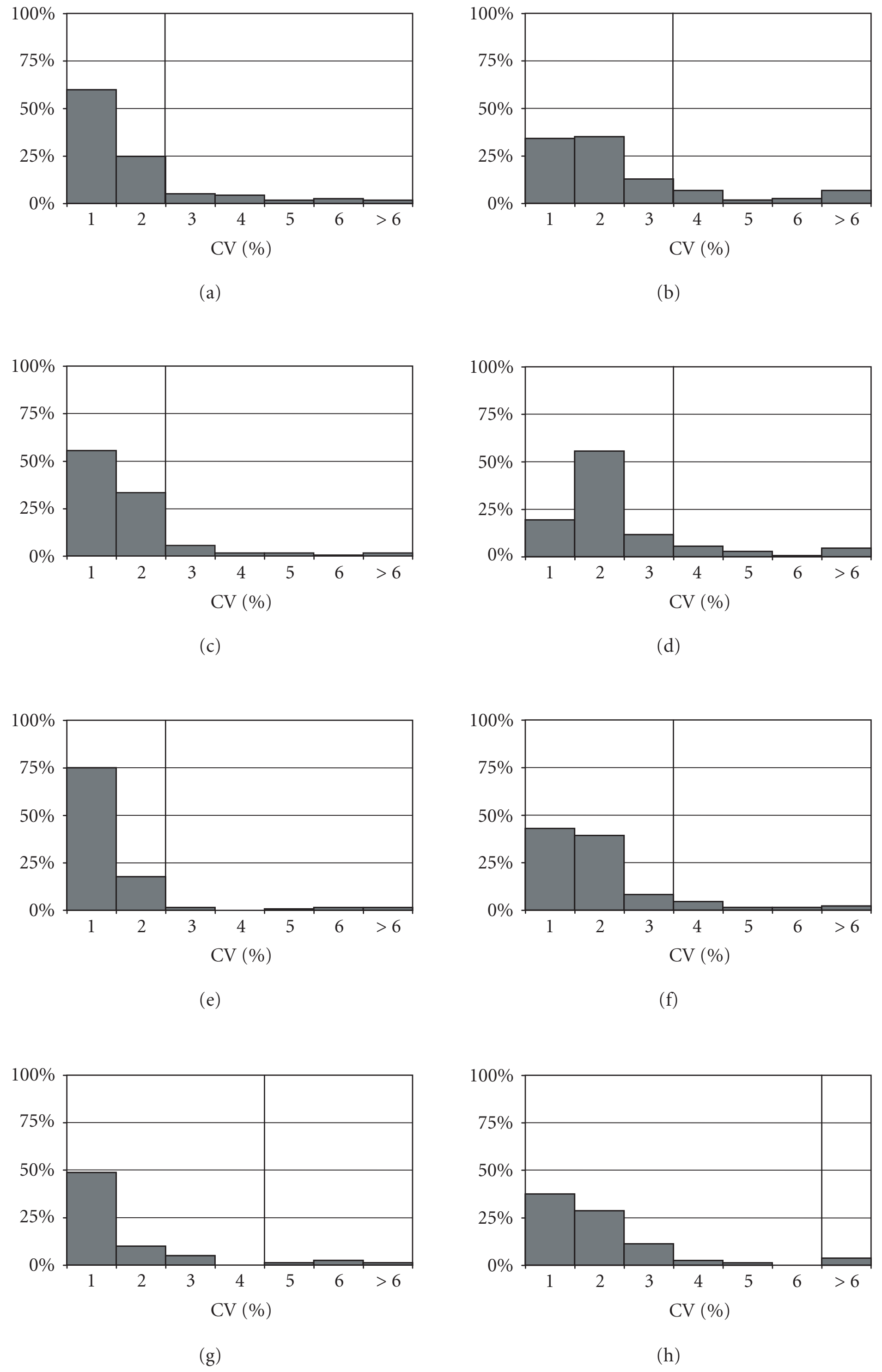

Figure 2: Imprecision in a simulated routine run, distribution of batch ((a), (c), (e), (g)) and random ((b), (d), (f), (h)) CVs for different analyte groups. (a), (b) Enzymes (117 CVs, 9158 results, 13 analytes). (c), (d) Substrates (180 CVs, 16165 results, 15 analytes). (e), (f) Electrolytes (136 CVs, 15006 results, 8 analytes). (g), (h) Proteins, TDMs (80 CVs, 2351 results, 27 analytes).

higher (average less than 1%) than the reference, as expected, but the differences were within the acceptance limits as defined by the study participants. Most deviations from the acceptance limits were due to expected causes such as analyte instability or low analyte concentration of the sample. One hardware problem, leaks in reagent sensor connectors, was

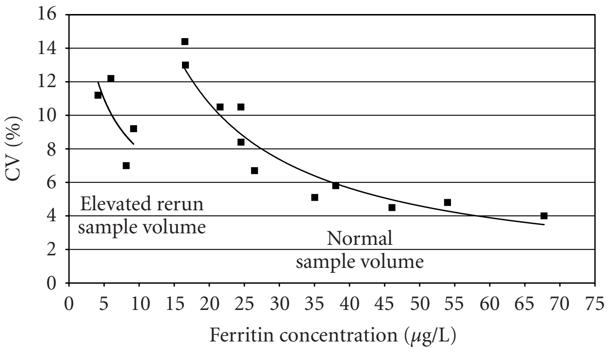

FIGURE 3: Functional sensitivity for the ferritin assay.

detected as a result of CVs exceeding the acceptance limits. (This problem was subsequently remedied.) At all laboratories, MODULAR handled "provocations" as designed. For example, when reagents were depleted while running, MODULAR switched to another bottle of equivalent reagent, either on the same module or on another available module; if no other reagent was available, MODULAR saved the samples in the rerun queue and alerted the operator to the problem, allowing for efficient resolution. When samples with insufficient volume (or empty sample cups) were encountered, MODULAR detected the problem, alerted the operator, but continued running the remainder of its workload without incident. MODULAR processed reruns automatically, diluting samples or decreasing sample volume if needed, according to user-defined limits and application technical limits.

The practicability [11] of MODULAR was compared to the existing situation in each laboratory using a questionnaire. The ratings from all participating laboratories for hardware, software, and lab integration are shown in Table 8. Higher ratings (7 to 10) were given more frequently for MODULAR than for the existing laboratory situation.

In Figure 5, the configurations of the MODULAR systems at three representative study sites are displayed, along with the distribution of the test requests per analyte performed by each module. Note that each site configured its system differently. As shown, lab A ran a total of 31 tests on MODULAR; lab B, 30 tests; lab C, 40 tests. Lab A used 12 of 16 D-module channels with 11 analytes duplicated on D- and P-modules while lab B used all 16 D-module channels with 6 analytes duplicated on D- and P-modules. Lab C, a double P-module configuration, assigned 28 of 37 tests to both modules. Many common chemistry tests were on all three systems, but several analytes were unique to each site. Lab A included lactate, haptoglobin, and cholinesterase; lab B included urine/CSF protein, haemoglobin A1c, and lipase; lab C included a variety of specific proteins.

To compare the effectiveness of MODULAR versus a combination of a Roche/Hitachi 747 plus a Roche/Hitachi 917, one operator from lab A performed the identical workload (a typical 16-hour period's work) in real time. As shown in Figure 6, 90% of 141 five-position racks (602 samples) run on MODULAR had rack-processing times (sample rack placement on MODULAR to results available) of less than 30 minutes. In contrast, the comparable figures for the Roche/Hitachi 917 and the 747 were 84% (27 of 32 racks) and 69% (66 of 96 racks), respectively.

Figure 7 displays the sample processing time (sample bar code registration on MODULAR to results available) at site B as the actual laboratory workload was performed. The spikes in the graph, corresponding to longer sample processing times (approximately 30 minutes), were associated with automatically rerun samples. Detailed analysis of rerun tests from approximately 3000 test requests run on fresh randomly selected samples showed that roughly 30 (1%) of the tests were rerun. Of these, 21 (70%) were related to lab policy (e.g., critical values that laboratories have traditionally repeated before reporting), and 9 (30%) were caused by analytical limitations (including dilutions, error codes, etc.).

TABLE 6: Analytical ranges, manufacturer claims.

| Analytes | Module | Units | Manufacturer claim |
|---|---|---|---|
| *Enzymes* | | | |
| AST | D/P | U/L | 800 |
| CK | D/P | U/L | 2300 |
| *Substrates* | | | |
| CHOL | D/P | mmol/L | 21 |
| CHOL-HDL | P | mmol/L | 4 |
| CREA J | D/P | μmol/L | 2200 |
| GLU | D/P | mmol/L | 42 |
| TP | D/P | g/L | 150 |
| UA | D/P | μmol/L | 1500 |
| *Proteins* | | | |
| CRP | D/P | mg/L | 3–240 |
| FERRI | P | μg/L | 15–800 |
| IGA | P | g/L | 0.5–8 |
| IGG | P | g/L | 3–35 |
| IGM | P | g/L | 0.25–6.5 |
| MYO | P | μg/L | 3–560 |
| *Electrolytes* | | | |
| CA | D/P | mmol/L | 5 |
| $CO_2$ | D/P | mmol/L | 45 |
| FE | D/P | μmol/L | 180 |
| CL | ISE | mmol/L | 250 |
| K | ISE | mmol/L | 100 |
| NA | ISE | mmol/L | 250 |
| *Urine* | | | |
| A1M_U | P | mg/L | 2–200 |
| ALB_U | P | mg/L | 3–400 |
| CA_U | P | mmol/L | 13 |
| CREAenz | P | μmol/L | 35500 |
| CREA J_U | P | μmol/L | 57500 |
| PHOS_U | P | mmol/L | 92 |
| UA_U | P | μmol/L | 16000 |
| UREA_U | P | mmol/L | 1300 |
| CL_U | ISE | mmol/L | 250 |
| K_U | ISE | mmol/L | 100 |
| NA_U | ISE | mmol/L | 250 |

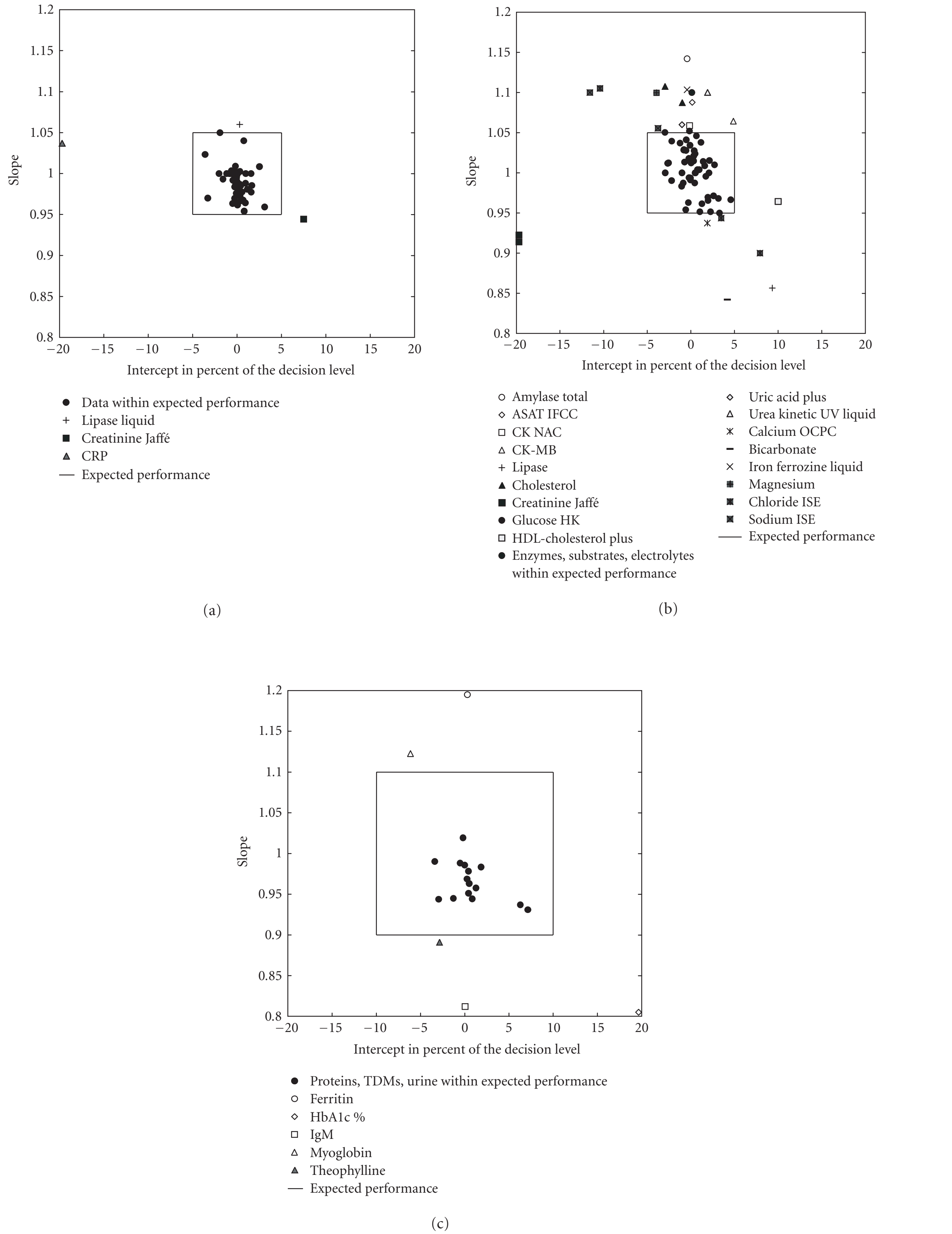

FIGURE 4: Method comparisons—summary of slope and intercept (a) D-module versus P-module, 57 comparisons, (b) enzymes, substrates, and electrolytes (P-module versus routine method, 72 comparisons), (c) proteins, TDMs, and urine analytes (P-module versus routine methods, 20 comparisons).

Table 7: Method comparisons exceeding the acceptance limits.

| Analyte | Unit | Lab | Regression analysis | | | Comment |
|---|---|---|---|---|---|---|
| | | | Slope | Intercept | md (95) | |
| Amylase total | U/L | 1 | 1.14 | −0.96 | 2.97 | X = UV-method |
| AST IFCC w/o PYP | U/L | 4 | 1.09 | 0.06 | 2.52 | X = optimised (DGKC) method |
| CK NAC | U/L | 13 | 1.06 | −0.29 | 5.76 | One of five labs, only on P-module, not on D-module, calibration effect |
| CK-MB | U/L | 2 | 1.06 | 1.17 | 25.97 | Only one lab, high scatter above 50 U/L |
| Lipase | U/L | 1 | 0.86 | 5.60 | 19.79 | X = method from Sigma. Similar results described in [14] |
| Cholesterol | mmol/L | 4<br>2 | 1.09<br>1.11 | −0.05<br>−0.15 | 0.19<br>0.16 | Two of five labs, download experiments yielded slopes from 0.94 to 1.06 |
| Creatinine Jaffé | $\mu$mol/L | 1<br>4<br>2 | 0.92<br>0.91<br>0.94 | −23.08<br>−24.96<br>8.64 | 15.32<br>20.75<br>16.68 | Lab 1 + 4: no compensation by an absolute term of 27 $\mu$mol/L during calibration |
| Glucose HK | mmol/L | 13 | 1.10 | 0.01 | 0.63 | One of five labs, download experiment yielded a slope of 1.04 |
| HDL cholesterol | mmol/L | 2 | 0.96 | 0.09 | 0.05 | At medical decision level (0.9 mmol/L) methods differ by about 6% |
| Uric Acid | $\mu$mol/L | 13 | 1.06 | −4.28 | 8.25 | At medical decision level (340 $\mu$mol/L) methods differ by 4.7% |
| Calcium | mmol/L | 3 | 0.94 | 0.05 | 0.15 | Calibration effect (stability) on the routine instrument |
| Bicarbonate | mmol/L | 13 | 0.84 | 1.87 | 1.99 | Analyte instability. Comparison must be performed at the same time |
| Iron | $\mu$mol/L | 1 | 1.10 | −0.12 | 0.80 | X = Cobas Integra 700, difference in standardisation, correction done |
| Chloride ISE | mmol/L | 4<br>13<br>2 | 0.94<br>0.90<br>1.11 | 3.67<br>8.40<br>−11.04 | 4.76<br>2.49<br>3.15 | Between 80 and 130 mmol/L the methods differ less than 5% |
| Sodium ISE | mmol/L | 13<br>3 | 1.10<br>1.06 | −16.80<br>−5.48 | 2.19<br>5.46 | Between 120 and 180 mmol/L the methods differ less than 5% |
| Magnesium | mmol/L | 13 | 1.10 | −0.04 | 0.055 | X = calmagite method, MODULAR xylidyl blue method traced back to AAS |
| Ferritin | mg/L | 2 | 1.19 | 1.17 | 73.33 | X = method from Beckman Access, LPIA method correlates well to the Enzymun and Elecsys method [15] |
| HbA1c% | % | 4 | 0.81 | 1.53 | 0.52 | X = Diamat HPLC method, refer to [16] |
| IgM | g/L | 4 | 0.81 | 0.08 | 0.14 | X = Cobas Integra 700 turbid. method, recently compared versus a nephelometric method yielding 20% lower results |
| Theophylline | $\mu$mol/L | 4 | 0.89 | −3.18 | 6.96 | X = FPIA method |
| Myoglobin | $\mu$g/L | 2 | 1.12 | −4.67 | 27.02 | Different standard sets on MODULAR and routine instrument |

Table 8: Questionnaire results as percent of total responses. Rating 1–3 = suggests improvement needed, 4–6 = meets lab requirements, 7–10 = exceeds lab requirements.

| Rating | Hardware (36 questions) | | | Software (75 questions) | | | Lab integration (77 questions) | | |
|---|---|---|---|---|---|---|---|---|---|
| | 1–3 | 4–6 | 7–10 | 1–3 | 4–6 | 7–10 | 1–3 | 4–6 | 7–10 |
| MODULAR | 8 | 47 | 45 | 3 | 43 | 54 | 6 | 44 | 50 |
| Current analysers(s) | 6 | 63 | 31 | 10 | 62 | 28 | 8 | 62 | 30 |

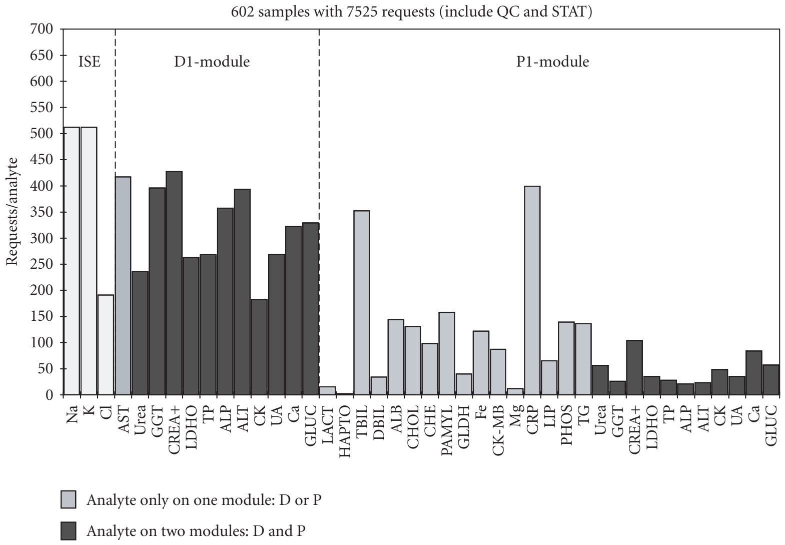

(a)

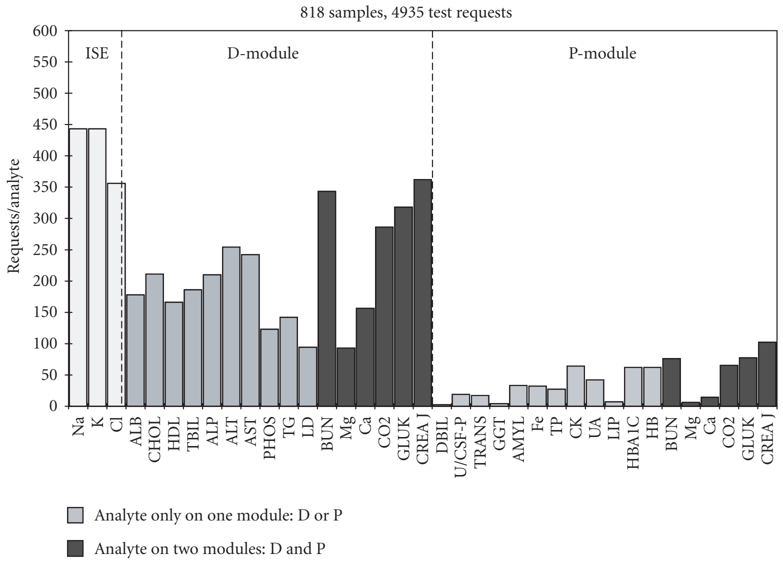

(b)

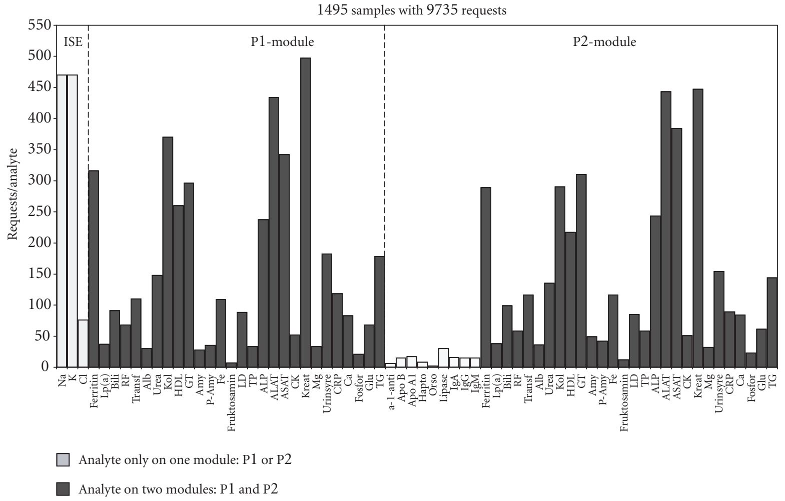

(c)

Figure 5: Distribution of test requests per analytes and module for (a) laboratory A, (b) laboratory B, (c) laboratory C.

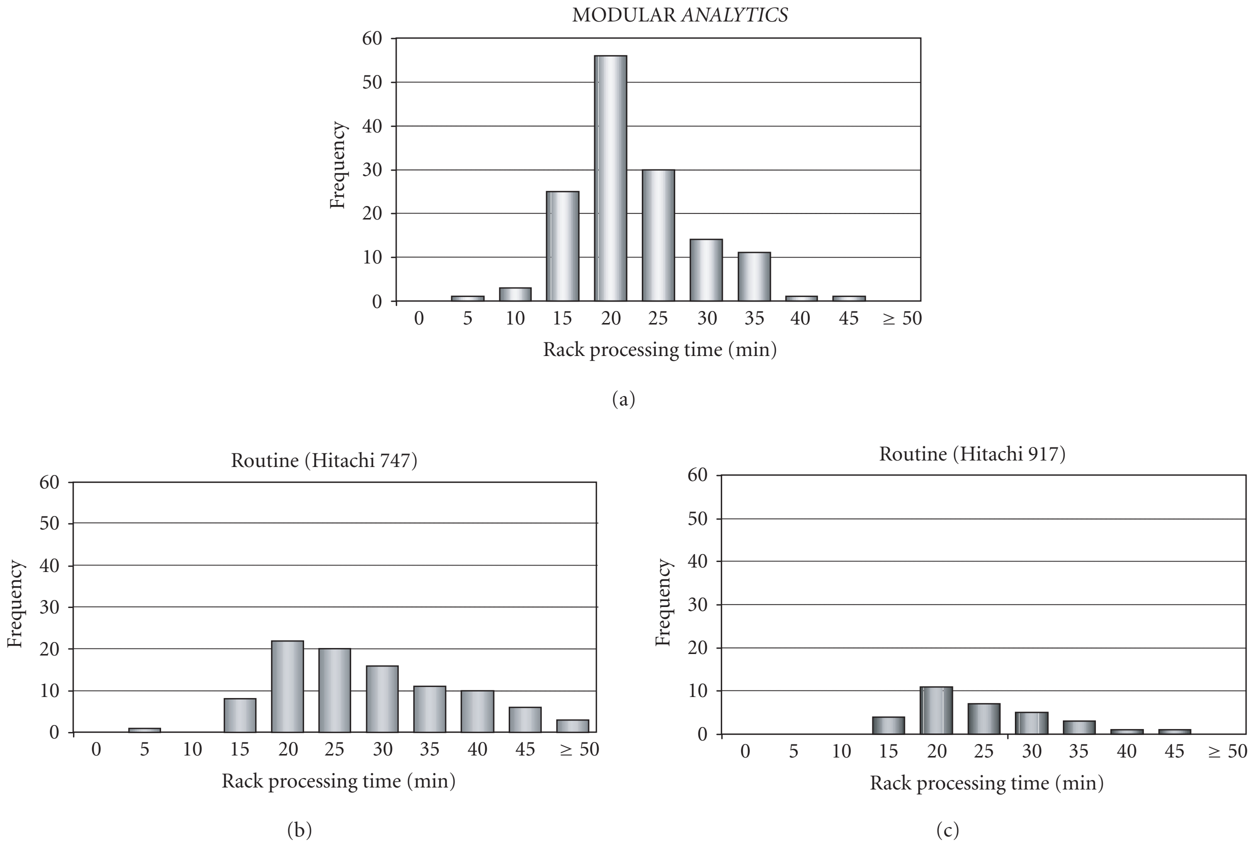

Figure 6: Rack processing time for MODULAR versus Roche/Hitachi 747 and Roche/Hitachi 917, laboratory A.

The reruns were all performed without operator intervention.

Lab B ran its workload in two different ways: as a single large batch (818 samples, 4935 tests) over 175 minutes simulating a commercial laboratory setting and as multiple smaller batches over about five hours representing a hospital central laboratory. Both types of situations were easily managed and completed without incident. To test the effectiveness of the STAT port, lab B introduced STAT samples while the system was in operation processing the normal morning workload. Table 9 provides the details on how the STAT samples were processed. In both cases, all 5 samples, with varying test requests as indicated, were completed within 13 minutes.

Figure 8 presents the throughput for the double P-module configuration from lab C. Continuous loading of 1495 samples with 9735 test requests (from 40 test methods) resulted in a throughput of about 250 samples per hour.

Routine daily maintenance required a total of 40 minutes (40–60 minutes if reagent preparation was included). However, as noted in Table 10, combining software features of parallel module maintenance, single module maintenance during operation, and automatically linked maintenance functions, lab B could perform maintenance such that the instrument was totally unavailable for only 13 minutes; during the other 27 minutes of maintenance, ISE tests plus one of the two modules were available to perform analyses.

### 4. DISCUSSION

The overall performance of MODULAR met (and, in some areas, exceeded) the needs and expectations of laboratory personnel. Expected performance criteria were established to help screen and manage the vast amounts of data generated. In almost all cases where some of the analytical methods did not meet preset expected performance criteria, the problems did not occur in all laboratories, and the methods met the manufacturer's claims. Furthermore, on careful review, none of the apparent shortcomings were deemed clinically significant.

### 4.1. *Imprecision*

For the electrolytes and the substrates, the within-run CVs for the results of both the control materials and human specimens were well within the acceptance limits. Routine simulation experiments revealed that the within-run CVs were systematically slightly better for batch analysis than those performed in random mode. The differences in CVs of these two modes, however, were within the acceptance limits. It can be expected that the imprecision obtained on a MODULAR system is higher than on a single analyser since the results could be generated on different analytical units, each requiring separate calibration.

Table 9: STAT sample processing during morning run (lab B). (The bold words denote **P-module**, the regular words denote D-module, and the underlined words denote ISE-module.)

| Rack | Samples (requests over three modules) | Time (on analyser to last result) |
|---|---|---|
| | 1 = **BUN**, **CREA**, Na, K | |
| | 2 = ALP, ALT, **AMYL**, AST, BUN, $CO_2$, CREA, **DBIL**, TBIL, Na, K, Cl | |
| 1 | 3 = **BUN**, **CREA**, Na, K | 13 min |
| | 4 = **BUN**, **CK**, **$CO_2$**, **CREA**, Na, K, Cl | |
| | 5 = **BUN**, **CK**, **$CO_2$**, **CREA**, Na, K, Cl | |
| | 1 = CA, PHOS, Na, K | |
| | 2 = CA, PHOS, Na, K | |
| 2 | 3 = **BUN**, Na, K | 13 min |
| | 4 = **BUN**, **$CO_2$**, **CREA**, **GLU**, Na, K, Cl | |
| | 5 = ALB, ALP, ALT, AST, **CK**, LD, TBIL, Na, K | |

The total imprecision obtained on a MODULAR system was expected to be equivalent to the combination of single analysers. The total variance can be estimated in a so-called nested design by

$$SD_{\text{MODULAR}} = \sqrt{sd^2_{\text{MODULE}} + sd^2_{\text{RUN}} + sd^2_{\text{REP}}}, \quad (1)$$

where REP denotes repetition.

A difference of ±5% between two modules was deemed acceptable. It has previously been shown that this was a realistic and achievable goal [18] for the earlier generations of Roche/Hitachi analyser. Based on the data from the drift experiment, we ascribe the relatively high bicarbonate between-day CV (7.2%) to analyte instability rather than method imprecision.

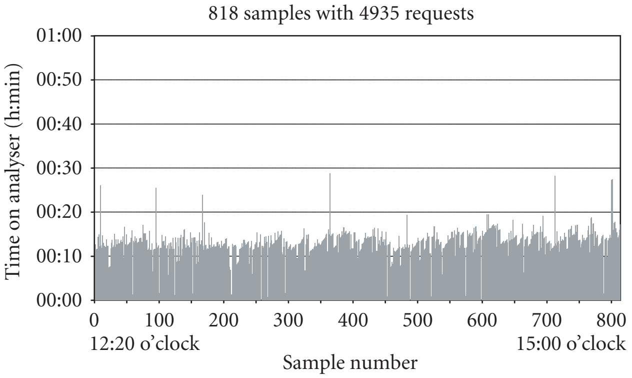

Figure 7: Sample processing time including automatic reruns (from bar code reader registration to result), laboratory B.

### 4.2. *Functional sensitivity*

Reliable measurements at high as well as low plasma ferritin levels are important for clinical decision making. Ferritin could be determined down to the manufacturer-specified limit of 15 $\mu$g/L, using the routine application on MODULAR; the CV at this concentration was 14%. However, if the concentration is less than 15 $\mu$g/L, MODULAR does an automatic rerun with increased sample volume. This enabled the functional sensitivity to be extended to less than 5 $\mu$g/L. This means that the ferritin assay can be used confidently to diagnose iron deficiency.

### 4.3. *Analytical range limits/interferences*

The acceptance criteria for linearity of the measuring range were fulfilled for all analytes. For standard spectrophotometric methods, the most frequent sources of interference are haemolysis, hyperbilirubinaemia, and lipaemia (turbidity). Of note is the fact that MODULAR, like its predecessors in the Hitachi line, is capable of estimating the level of these interferents from the measurement of "serum indices," an additional test based on absorbance readings taken at multiple wavelengths of each sample diluted with saline [19]. In the case of AST, LDH, and K, the positive interference in haemolytic specimens is not due to haemoglobin itself, but due to these substances being liberated from erythrocytes during haemolysis. In a similar way, the increase in iron with increasing haemolysis is not a true interference but a reflection of the haemoglobin-bound iron. Even though the CK reagent contains inhibitors of adenylate kinase (AMP and diadenosine pentaphosphate), at high enough levels of haemolysis (120 mmol/L), this inhibition is overcome and the apparent CK activity increases.

### 4.4. *Accuracy*

As noted in Results, accuracy of the methods on MODULAR was established in three different ways. In all five laboratories, the recoveries of both control materials for the 17 methods tested were within 5% of the assigned values. Second, for the certified reference materials (NIST and CRM), all but two of the results (both cholesterol) were within 5% of the assigned value. In the case of cholesterol, the higher than expected recoveries were probably due to the value assigned to the calibrator. When repeated with a new calibrator, the recoveries of the NIST materials went from 99–108% to 96–103% (all within the 95–105% acceptance criteria).

Third, 65 out of 92 method comparisons performed versus existing non-MODULAR methods gave slopes and intercepts that were within the acceptance limits. For 7 analytes, the comparison methods were intrinsically different (e.g., different substrates for amylase), which explains the higher deviations from, and higher scatter around, the regression line. The deviations of the remaining 20 methods were caused by different standardisation, calibration effects, analyte instability or narrow range of data points; detailed explanations are given in Table 7.

### 4.5. Functionality and practicability

When evaluating new analytical systems, it is important to determine whether the new systems can achieve their potential in real operating laboratories, where a number of different variables come into play. The number of interactions increases substantially as the number of different chemistry test methods run on an analyser increases. It is difficult, if not impossible, to detect all such possible combinations utilising traditional evaluation methods, but the opportunity to detect (and correct) such situations increases greatly when the new system is evaluated under routine laboratory conditions, as we did in the routine simulation experiments [10]. For example, the occasional leaks in the reagent sensor connectors that were noticed during these experiments (as deviant results) were repaired by a hardware modification.

According to the questionnaire results, MODULAR met laboratory requirements and offered an improvement over the current laboratory situation in the area of lab integration as well as in hardware and software related attributes. The main advantages of the system cited were: efficiency gained through workstation consolidation and automatic rerun, ease of use and training, high throughput combined with high reliability of results, and versatility offered by an extensive test menu and the ability to expand the system. The main perceived shortcomings mentioned were the inability to reload reagent during operation (advantageous, even if not entirely necessary, for most labs), the need to put a module back into service more quickly once offline troubleshooting was completed, and the desire for easier access to internal parts for operator maintenance. Shortly after the evaluation was completed, the first two shortcomings were addressed by software changes. An additional point of discussion was the potential need for some back-up analytical system if the track, or another central part, of MODULAR failed. The need for such back-up systems is lab-specific and depends on service levels offered, availability of other instrumentation in the central lab, access to satellite labs, etc. However, based on the experience from this multicentre trial we can say with confidence that the probability of a central failure of MODULAR is very low.

Many discussions on laboratory automation today focus on workstation consolidation—combining a number of traditionally distinct methodologies on a single analyser [20, 21, 22]. For example, in one study, seven workstations were reduced to two "multi-functional" analysers, offering photometry, turbidimetry, ion selective electrodes, and fluorescence polarisation, with concomitant reductions in turnaround time, errors and sample splitting [23]. MODULAR offers this kind of workstation consolidation, with over 100 methods available (corresponding to more than 80 analytes), encompassing electrolytes, routine chemistry testing, specific proteins, TDM, toxicology, and other homogeneous immunoassays. Furthermore, MODULAR provides additional flexibility and capabilities. MODULAR allows STAT samples to be processed, for the full repertoire of testing, while processing its regular workload. This may enable some laboratories to incorporate separate STAT laboratories into a single MODULAR workstation in their main laboratories. Additionally, if it turns out that one has initially underestimated the test repertoire or throughput required, one has the flexibility of adding modules to the system as needed.

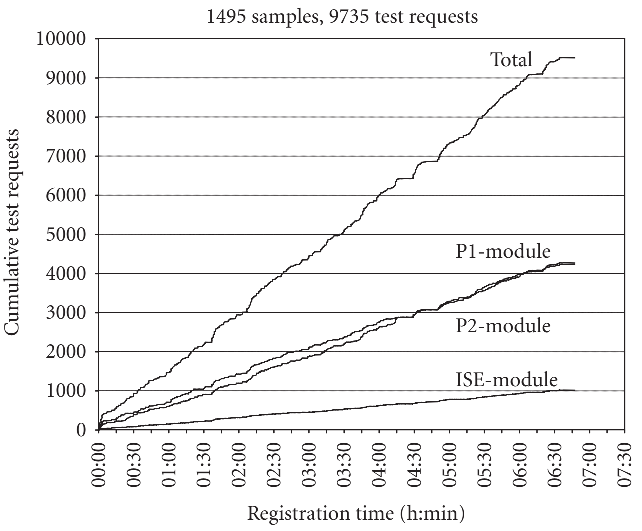

FIGURE 8: Throughput for large batch workload (ISE-, P1-, P2-module configuration), laboratory C.

Perhaps a more important criterion for evaluating a system's effectiveness today is the time it takes to complete its analyses. What laboratories really need to consider is the time it takes to get results back to the ordering physician. Looking at the data from lab A, we know that, for more than 90% of its samples, the rack processing time (i.e., the time from when the operator placed the 5 samples on MODULAR until the analyses were completed) was less than 30 minutes. In this regard, MODULAR met or exceeded the laboratories' requirements. The time to results for samples with requests on both the 917 and 747 is in fact even longer than presented here because only the sum of the processing times without the time for transfer between individual systems was considered. The throughput of MODULAR was quite acceptable whether one was running the system as a commercial laboratory (simulated as one large-batch run in this study), or as a hospital central laboratory (in multiple smaller batches), or as samples arrive in the laboratory. Lab C was able to meet its expectations and requirements for workflow, too, even though it processed a large number of samples with many different analytes. Their choice of a P+P configuration, with duplication of 28 analytes, enabled them to process samples effectively in serial, parallel, and serial/parallel fashion.

However, when considering the time it takes to complete analyses on a modern system, one must also consider the time it takes to do reruns, to process STAT samples, and to resume testing when reagents need to be replenished unexpectedly. In Figure 6 the samples whose processing time is

Table 10: Daily maintenance procedure maximising operating time (lab B).

| Maintenance period | Maintenance type | Analyser status | Available tests | Time required (min) |
|---|---|---|---|---|
| 1 | Parallel maintenance[1] | Standby | 0 | 13 |
| 2 | D-module maintenance[2] | P-module operational | ISEs + 17 | 15 |
| 3 | P-module maintenance[3] | D-module operational | ISEs + 16 | 10 |

1 includes D-module bath exchange, ISE- and P-module air purge, ISE prime, sample probe clean and adjust, ISE calibration.
2 includes air purge, mechanical check, reagent prime, photometer check, adjust stirrers, prime new reagents, clean rinse nozzles.
3 includes P-module bath exchange, photometer check, clean and adjust stirrers and reagent probes, clean rinse nozzles.

longer than the typical 20 minutes are seen as peaks. These were actually automatic rerun tests which represent one of the major advantages of MODULAR software, internal track system connecting modules, and input, holding, and output buffers. By automatically processing the rerun tests, the system does, in a much more efficient way, what a human operator would normally be required to do, freeing the operator and allowing the system to optimise sample processing. Similarly, when reagents are depleted, MODULAR automatically uses equivalent reagents, even if it means shunting samples to another module; when STAT samples are introduced they are processed according to computer-optimised scheduling. The combination of the fast, efficient, hands-off sample processing with the large repertoire of tests makes MODULAR a very effective system. With the ISE + D + P module configuration, the system can offer as many as 63 different chemistry tests simultaneously. Thus, the typical sample processing times of 20 minutes and rack processing times of 30 minutes reflect a potentially very large proportion of a laboratory's total work and most samples' complete test requests. That is, when samples arrive in the MODULAR output buffer, they are likely totally completed. Laboratory B calculated that MODULAR, even without its maximised test repertoire, covered more than 90% of its test requests in the chemistry laboratory.

In conclusion, MODULAR performed well technically and operationally during the evaluation. The workload and the workflow studies showed the ability of MODULAR to handle the workload and workflow of multiple instruments with over 100 methods. The total testing time on MODULAR was faster than the individual analysers by 30 minutes. Thus, MODULAR begins to constitute a "third-generation analyser," whose features include tracks that move samples between modules and a computer that handles scheduling and other automated tasks [22].

Further progress toward total consolidation has occurred since the completion of this evaluation. For immunochemistry laboratories, Roche introduced MODULAR *ANALYTICS* E170 module (E170), based on the well-established electrochemiluminescence methodology of the Elecsys analyser [24]. However, major advancement occurred when Roche launched MODULAR *ANALYTICS* Serum Work Area (INTEGRATED MODULAR *ANALYTICS* in the US market), which allowed E170 modules and ISE-, D-, and P-modules to be combined on one platform, thereby consolidating heterogeneous immunoassays (E170) with the electrolyte, substrate, enzyme, and homogeneous immunoassay methods described in this paper.

### ACKNOWLEDGMENTS

The authors wish to thank all their coworkers in the respective laboratories and departments participating in the study for their excellent support. For the studies described in this report, Roche Diagnostics lent all evaluation sites the MODULAR *ANALYTICS* system and a personal computer with the evaluation software CAEv. In addition, Roche Diagnostics provided all necessary reagents, calibrators, controls, and disposables.

### REFERENCES

[1] R. S. Seaberg, R. O. Stallone, and B. E. Statland, "The role of total laboratory automation in a consolidated laboratory network," *Clin Chem*, vol. 46, pp. 751–756, 2000.

[2] R. Haeckel, E. W. Busch, R. D. Jennings, and A. Trucheaud, Eds., *Guidelines for the Evaluation of Analysers in Clinical Chemistry*, vol. 3 of *ECCLS Document*, Beuth Verlag, Köln, Berlin, Germany, 1986.

[3] NCCLS Evaluation Protocols, National Committee for Clinical Laboratory Standards, Villanova, Pa, USA, 1992.

[4] J. T. Nicoloff and C. A. Spencer, "The use and misuse of the sensitive thyrotropin assays," *J Clin Endocrinol Metab*, vol. 71, no. 3, pp. 553–558, 1990.

[5] W. Bablok, "Range of linearity," in *Evaluation Methods in Laboratory Medicine*, R. Haeckel, Ed., pp. 251–258, VCH, Weinheim, Germany, 1993.

[6] H. Passing and W. Bablok, "A new biometrical procedure for testing the equality of measurements from two different analytical methods," *J Clin Chem Clin Biochem*, vol. 21, pp. 709–720, 1983.

[7] P. M. G. Broughton, A. H. Gowenlock, J. J. McCormack, and D. W. Neill, "A revised scheme for the evaluation of automatic instruments for use in clinical chemistry," *Ann Clin Biochem*, vol. 11, pp. 207–218, 1974.

[8] R. Haeckel, "Recommendations for definition and determination of carry-over effects," *J Autom Chem*, vol. 10, pp. 181–183, 1988.

[9] M. R. Glick, K. W. Ryder, and S. A. Jackson, "Graphical comparisons of interferences in clinical chemistry instrumentation," *Clin Chem*, vol. 32, pp. 470–475, 1986.

[10] W. Bablok and W. Stockmann, "An alternative approach to a system evaluation in the field," *Quim Clin*, vol. 14, p. 239, 1995.

[11] W. Stockmann, W. Bablok, W. Poppe, P. M. Bayer, F. Keller, and C. R. Schweiger, "Criteria of practicability," in *Evaluation Methods in Laboratory Medicine*, R. Haeckel, Ed., pp. 185–201, VCH, Weinheim, Germany, 1993.

[12] P. Bonini, F. Ceriotti, F. Keller, et al., "Multicentre evaluation of the Boehringer Mannheim/Hitachi 747 analysis system," *Eur J Clin Chem Clin Biochem*, vol. 30, no. 12, pp. 881–899, 1992.

[13] W. Bablok, R. Barembruch, W. Stockmann, et al., "CAEv—a program for computer aided evaluation," *J Autom Chem*, vol. 13, pp. 167–179, 1991.

[14] W. Junge, K. Abicht, J. Goldman, et al., "Multicentric evaluation of the colorimetric liquid assay for pancreatic lipase on Hitachi analyzers," *Clin Chem Lab Med*, vol. 37, no. special supplement, p. 469, 1999.

[15] G. Klein, W. Duchna, G. Hafner, et al., "International multicenter evaluation of Elecsys® ferritin on the Elecsys® 2010 and 1010 analyzers," *Clin Chem*, vol. 44, suppl, p. A47, 1998.

[16] D. L. Bakkeren, P. Bonvicini, M. Buxeda, et al., "Multicenter evaluation of an improved immunoturbidimetric assay for the determination of HbA1c on clinical chemistry analyzers," *Clin Lab*, vol. 45, pp. 123–137, 1999.

[17] W. Bablok, R. Haeckel, W. Meyers, and W. Wosniok, "Biometrical methods," in *Evaluation Methods in Laboratory Medicine*, R. Haeckel, Ed., pp. 203–241, VCH, Weinheim, Germany, 1993.

[18] Z. Zaman, N. Blanckaert, and L. Sneyers, "Inter-instrument transferability of results from different BM/Hitachi analyzers," *Proceedings Ass. Clin. Biochem.*, p. 82, 1993.

[19] M. R. Glick, K. W. Ryder, D. H. Vroon, B. E. Masters, and O. Sonntag, "Practical uses of serum indices to reduce errors from lipemia, icterus, and hemolysis," *Clin Chem*, vol. 36, no. 6, p. 1008, 1990.

[20] G. E. Hoffmann, "Concepts for the third generation of laboratory systems," *Clin Chim Acta*, vol. 278, no. 2, pp. 203–216, 1998.

[21] K. Luczyk, "Preparing the lab for the year 2001: workstation consolidation," *MLO Med Lab Obs*, vol. 29, no. 3, pp. 42–44, 1997.

[22] A. Mira and C. Lehmann, "Workflow analysis an international tool: cost reduction while retaining personnel," *Clin Lab Manage Rev*, vol. 13, no. 2, pp. 75–80, 1999.

[23] P. J. Brombacher, G. J. Marell, and L. W. Westerhuis, "Laboratory work flow analysis and introduction of a multifunctional analyser," *Eur J Clin Chem Clin Biochem*, vol. 34, no. 3, pp. 287–292, 1996.

[24] K. Erler, "Elecsys® immunoassay systems using electrochemiluminescence detection," *Wien Klin Wochenschr*, vol. 110, suppl 3, pp. 5–10, 1998.

